# On the Treatment of Intermitting Fever

**Published:** 1844-08

**Authors:** Austin Flint

**Affiliations:** Professor of Institutes, and Theory and Practice of Medicine, in the Rush Medical College; Buffalo


ILLINOIS

MEDICAL & SURGICAL JOURNAL.

VOL. I. AUGUST, 1844. NO. 5.

*On the Treatment of Intermitting Fever.* By Austin Flint, M. D., Professor of Institutes, and Theory and Practice of Medicine, in the Rush Medical College.

The subject of intermitting fever occupies so small a portion of periodical medical literature, in comparison with the frequency of the disease in many extensive sections of our country, that in communicating observations which are supposed to possess novelty, it may easily happen that we are only stating facts with which a large number of our professional brethren are already familiar, and which, to a considerable extent, have had their legitimate practical influence. This may be the case with the points upon which it is my purpose to present the results of my experience in the following remarks, viz:—the exhibition of quinia in larger doses than are generally directed by standard writers, and without those preliminary and precautionary measures, which have been considered very important to be observed in the treatment of the disease. When I found cause to modify my own views concerning the use of this specific, I was not aware that others had been led by their observations upon similar ground. I have, however, since ascertained, that the ideas which I shall offer have no claims to originality. In the late work of Professor Elliotson, (which I have recently seen,) the same principles are recommended which circumstances had led me to entertain. Since my attention has been particularly directed to this subject, I have also seen similar views in the various medical journals. Supposing, nevertheless, it to be highly probable that ~~there is,~~ (as they appear to me,) in many instances, improved methods of managing the disease ~~which~~ have not been generally adopted, I submit the results of their application, to a considerable extent in my hands, as confirmatory testimony of their value.

I was first led to experiment upon the safety and expediency of prescribing the salts of quinia in large doses, in the autumn of 1839, while performing the duties of acting assistant surgeon at Poinsett Barracks, near this city. Several companies of the second artillery being transferred from Fort Gratiot, Michigan, to this port, intermitting fever was rife among the newly arrived troops. The majority of those sick with this disease not being received into hospital, owing to the limited accommodations at that time, the inconvenience of the patients coming from their quarters hourly, and the uncertainty of trusting the medicine to their own administration, suggested the trial of prescribing the whole quantity which it was desired should be taken during apyrexia, in one or two doses. Commencing with five grains, and repeating in twenty or thirty minutes, the amount was increased until twenty, thirty, and in one case, even forty grains were administered within a half hour. After satisfying myself not only of the safety, but, in many cases, the superiority of this plan of treatment, my usual course was to prescribe eight, ten, or fifteen grains, and if no unpleasant results followed, repeating it in from twenty to sixty minutes, whenever doubt was entertained concerning the ability of the patient to bear the whole quantity which I wished to administer during the apyrexia. But experience having shown that no inconvenience of any moment followed the exhibition of twenty or thirty grains within a short space of time, I was afterwards in the habit, in the majority of cases, especially when the patients were not retained in hospital, of prescribing, at once, these quantities.

At this period, I commenced keeping a record of the cases which fell under my observation, which I continued until seventy-one cases were collected. As I did not immediately adopt the method of large doses in private practice, forty-three only of this number will come under this class; and of these, thirty-six were soldiers.

It may be important to remark, that none of the patients were probably aware that they were taking medicine in an unusual quantity.

As the case in which the largest quantity of the sulphate of quinia was administered may possess particular interest, I present the details as recorded in my case book.

*Bailey*, seized with chill and rigors, about two minutes before his name was called to present himself for examination. Sulphate of quinia, twenty grains, was prescribed and administered immediately. In a few minutes, said that he felt better; chill and rigors, however, still continued. Directed repetition of the same dose, making forty grains. In a few moments, said he felt dizzy and nauseated. Gave him a bed in hospital. An hour afterwards examined him. He complained of deafness, dizziness, and nausea; did not vomit; pulse now much accelerated, moderately full; skin warm. Hospital steward reported the subsequent day, that deafness and dizziness continued for several hours. Found him on this morning with aspect bright, and said he felt quite comfortable; slept well. Two days afterwards he had had no recurrence of the paroxysms. No farther medicine was exhibited; desired to go to duty, and was returned accordingly.

It is an interesting subject of inquiry—at what period of the apyrexia may the specific be exhibited to exert the most efficiency? It is generally supposed, that the nearer, within a certain limit, to the time of the expected recurrence of the paroxysm it is administered, the greater will be the probability that the paroxysm will be averted, or its severity diminished. In the records of the preceding cases, I was not careful always to note this relation. I will state, however, that the usual course was to have it administered directly after its prescription, in the majority of cases, for the sake of convenience, without any reference to the period at which the patient expected a paroxysm to recur. Shortly before I was relieved of my attendance at the barracks, however, this point suggested itself, and I proposed to make it the subject of some observations, by exhibiting the quinia in a large dose at an early period of an intermission, and omitting it during the latter period. This was practised in four cases only, a brief account of which is as follows.

Case I.—Sulphate of quinia was administered in dose of fifteen grains, *ten* hours before the period when the paroxysm regularly would have occurred. The patient had no subsequent paroxysm. The same dose was repeated three successive days, and the patient discharged.

Case II.—Sulphate of quinia, ten grains, was administered *six* hours before time of paroxysm; the paroxysm was not prevented. The same dose, with the same interval, was repeated on the ensuing day; no paroxysm occurred on this day. This case was complicated with otitis.

Case III.—In this case, sulphate of quinia, fifteen grains, and after 30 minutes, ten grains, were administered *thirty-seven* hours previous to the time of the paroxysm. The paroxysm did not occur. No farther medicine was administered to the patient, and he was returned for duty on the seventh day. Seven days afterwards he returned with relapse. He was then bled ℥xii in the hot stage, and treated with ten grains morning, noon, and evening, for the first day; eight grains, morning, noon and evening, second day; six grains, morning, noon and evening, fourth day. No paroxysm afterwards occurred while on the sick report, but subsequently he relapsed.

Case IV.—In this case, twenty-five grains were administered 34 hours previous. Paroxysm was not prevented. The same dose was similarly administered on the day after the paroxysm. The paroxysm did not occur, and the patient was discharged on the fifth day. This case was complicated with catarrh and pain under the sternum on full inspiration. No other treatment than with the quinia was pursued.

Another inquiry connected with this branch of the subject possesses interest, viz.:—In how far will the quinia exhibited at the commencement of a paroxysm, tend to avert it, to mitigate its severity, or shorten its duration? This method has been recommended as the most efficient for the administration of the specific. And on the other hand, the late Dr. Eberle states, that in a single instance under his observation, it appeared to be followed by dangerous results. I made trial of this procedure in seven cases, one of which has been already presented.

The inferences to be drawn from which cases are, that sometimes the paroxysm may be arrested by exhibiting the sulphate of quinia during the cold stage, and its severity and duration diminished in other instances; but neither of these results ought to be confidently expected. It would seem that when the paroxysm is not strangled, the effects of the specific upon the subsequent continuance of the disease is not less than when administered in the apyrexia. In none of these cases, were any alarming or injurious effects produced. It is not, however, presumed to state these inferences as facts established from such a limited number of cases. A larger collection of observations, with reference to this subject, is much to be desired.

I proceed to present a few cases selected from those which have been treated in private practice. The two first are only interesting, from the fact that the subjects were females of delicate constitutions, and from the second case being complicated with gastric irritability, which rendered the exhibition of a tonic in small doses, frequently repeated, impracticable, but admitted of its administration in larger doses without inconvenience.

Case I.—Mrs. P., ætat. 25, constitution extremely delicate, was attacked with intermitting fever of quotidian type, which was speedily relieved by Carpenter's precipitated extract of bark, (an article which I have much used, and found to possess equal, if not superior virtue to the sulphate of quinia,) in doses of one grain hourly during the apyrexia. As soon as the paroxysms were interrupted she discontinued the medicine, contrary to advice. Several weeks afterwards she was again attacked with the disease of the tertian type. She had had three paroxysms previous to my being requested to visit her. Without preliminary treatment, sulphate of quinia, in doses of five grains morning, noon, and evening of the day of apyrexia, was prescribed. Visiting her in the evening, I ascertained, that mistaking the directions, she had taken the powders hourly, until the whole number written for (five) had been taken. She had thus taken twenty grains within five hours. No unpleasant effects were produced, excepting slight nausea, and some ringing in the ears, which it did not occur to her to attribute to the medicine. She had no recurrence of the paroxysms. She continued to take four grains daily for six days. Ten months have now elapsed, and this lady has experienced no return of the disease.

Case II.—Mrs. S., ætat. about 28, delicate constitution; after returning from a visit at Toledo, Ohio, was seized with intermittent fever of quotidian type. The paroxysms produced great prostration, complete anorexia, with restlessness and watchfulness during the apyrexia. Carpenter's extract was prescribed in doses of one grain in solution hourly. This occasioned so much nausea, that pills of the same, containing grs. iiss were substituted, during the next apyrexia. The same effect followed to such a degree that it was impossible for her to continue them. On the next day sulphate of quinia, five grains, repeated in 45 minutes, was prescribed. She took both powders with slight inconvenience, and the paroxysm of this day was much less severe. The next day she took five grains, and escaped the paroxysm. For three days subsequent she took grs. iiss, and rapidly recovered her appetite and strength. Several weeks afterwards this lady had severe intermittent neuralgia, which was cured by a resort to the same method.

An idea seems to prevail to a considerable extent, both among patients and physicians, that if the paroxysms be permitted to continue for a certain period, the liability to relapses is less than if immediately checked. The late Dr. Eberle advocated this idea. I have known repeated instances, in which patients have resigned themselves to an endurance of this distressing malady for a long period, under the impression that if it be allowed to "wear itself out," they would thereafter be secure against its return. My experience is directly opposed to these views. The cases most difficult to manage, and in which relapses have been most frequent, have been among those who, from necessity, or the erroneous idea just mentioned, have not early resorted to means of relief. And on the other hand, the cases in which I have earliest succeeded in interrupting the paroxysms, the immunity has been the most permanent. I have entertained the conjecture that careful attention in many of these cases which terminate in remittent fever, at their primary stages, would disclose periods of intermission, when the specific may be administered in large doses with entire safety, and arrest the progress of a disease which otherwise might become confirmed and continue for weeks.

*General remarks.*—The majority of the cases of intermitting fever which occur in this city, (Buffalo,) are contracted in other localities. The disease was formerly rife here, but is now confined in its origin to a few contracted districts; and in these, the quantity of malaria seems to be insufficient to induce an attack, excepting in those peculiarly predisposed, or in conjunction with other concomitant circumstances. From the commercial character of the place, however, and the general habit which prevails among the business portion of the community, of travelling more or less during the summer season, through the western states and territories, the occurrence of the disease is very frequent. We are also constantly in the way of observing it in returning emigrants and travellers of all classes.

Not having resided in any locality where the disease is endemic to a greater extent than here, I cannot speak of the success of remedial measures where the patient is exposed to the continued concentrated action of the morbific principle. I can state, however, that of a large number of cases, of a very miscellaneous character, which have fallen under my observation for the past five years in this place, in private practice and at the almshouse, as well as the military station located near the city, I cannot recall a single instance in which the paroxysms were not promptly interrupted whenever the patients could be made follow the directions given.

Unfortunately, a prejudice against the use of the invaluable specific for this disease, appears to prevail extensively throughout all malarious regions. It is thought to injure or undermine the constitution; and this deplorable error is diligently promoted by a host of unprincipled individuals, who are interested in the diffusion of various empirical nostrums, the potency of which is generally wholly derived from the very article which they endeavour to discredit. Such an impression pervading the common mind, affects the views of many physicians; they hesitate to employ the remedy with sufficient promptitude and boldness, and fearful of latent mischief, administer it with a degree of caution and delay, which serves to countenance and confirm that prejudice which is so much to be regretted. The preceding cases will, I trust, be sufficient to demonstrate that the sulphate of quinia may be exhibited in doses sufficiently large to ensure prompt relief without any fear of unpleasant consequences. Having employed it also in other affections, especially some forms of neuralgia, in doses equally large, and continued its use much longer than in any of the cases of intermitting fever which have been presented in this communication, I have satisfied myself that it may with entire safety be given in large doses, and repeated for a length of time, which it is presumed will be sufficient, in any case, to accomplish the end which is desired.

Many practitioners are deterred from the prompt exhibition of the quinia, by the impression that the system requires some preparatory processes for its reception; especially when there are present a thickly coated tongue, with other evidences of general disturbance of the secretory functions, and more particularly if gastric derangement is a prominent difficulty, the indefinite idea expressed by the term "bilious" is invoked, and regarded as an insuperable obstacle to its administration. I have been led to regard these symptoms as oftener the effects than purely the adventitious concomitants of the disease. In a large proportion of cases, we find there is a tendency to irritation of the gastro-duodenic mucous membrane, indicated by nausea, vomiting, anorexia, &c. This is increased and confirmed the longer the disease is permitted to continue. Under this view, the most rational policy is, manifestly, to strike at once at the *fons et origo* of the difficulty. There will be cases, it is true, where the specific will not be easily borne; but it will often be retained with slight inconvenience, on exhibiting a second dose, when the first was either rejected or created much irritation; and may, as we have seen, sometimes be borne well in a large dose, when it is insupportable in small quantities. At any rate, it is difficult to conceive in what manner the irritation of emetics and cathartics, in conjunction with the continued recurrence of the paroxysms, is to communicate a better state of preparation than had previously existed.

Other and more serious complications may sometimes exist, or their existence be suspected, when it may seem that the excitant properties of the quinia, in sufficient doses to effect a speedy cure, may expose the patient to more dangerous results than than would follow from the unchecked paroxysms. In such cases, the instructions of some standard authorities are, first, to relieve the concomitant affection by appropriate measures, and afterwards, the intermitting fever. We are to be guided, as it appears to me, in such cases, by the considerations which will arise from the following inquiry:—Will the excitant properties of the tonic tend more to exaggerate the accompanying disease, than the series of morbid actions which constitute the paroxysms of intermitting fever? I am disposed to think that the cases are very few in number, in which the true answer to this inquiry is affirmative. In my own experience, I have not, as yet, met with an instance in which I have hesitated to employ ample means to interrupt the paroxysms as promptly as possible; nor have I ever found reason to regret, in any instance, having pursued this course. Undue apprehensions on this score have, I suspect, been among the unfortunate influences of the doctrines of Broussais. Indeed, this author states, (Chronic Phlegmasiæ,) that in certain cases, "nothing in medicine presents more difficulties than the treatment of intermitting fevers." "On the one hand," he adds, "the series of vital actions which constitute the intermittent fever, may tend to produce inflammatory nucleii in the lungs;" and on the other hand, he presents cases to show, that the tonic medicine necessary to effect an immediate cure, may cause an incurable phlogosis of the stomach. I can hardly conceive of this latter result being produced, for I have often administered this remedy in considerable doses where the stomach was irritable, and after a short period of increase of the symptoms, have witnessed the irritability rapidly subside, so that subsequent doses were borne without any unpleasant effects. In such cases, if we were to shut out of view the existence of the intermitting fever, we might almost come to the conclusion, that the quinia was an appropriate remedy, in certain cases, for gastric irritability; and, assuredly, if it ever tends to produce phlogosis, we should presume that this tendency would be especially exerted when a state of great irritability was already present. The general excitant effects of the quinia, exerted upon the nervous and circulatory systems, certainly bear no comparison with the several stages of an intermittent paroxysm. In fact, in the majority of cases, when exhibited in moderate doses, these effects are hardly perceptible. Even when administered during the intense febrile excitement of the hot stage, (I have been informed by a highly intelligent medical friend who practised in a malarious district, and was in the habit of continuing the use of the specific throughout the whole of this stage,) the symptoms are not increased, nor any unpleasant effects produced. I cannot imagine, then, that the coexistence of even a well marked phlogosis of any organ beside the stomach itself, is, in reality, a sufficient obstacle in the way of the administration of this remedy. On the other hand, apprehending far more evil from the repetition of the paroxysms than from the excitant properties of the tonic, I should not hesitate, were a case of this kind to fall under treatment, to administer the remedy in doses sufficiently large to arrest the recurrence of the paroxysms as speedily as possible, employing, in conjunction, such depletory and revulsive measures, as the character of the local affection, under other circumstances, would demand. If the gastric mucous membrane itself were in a state of inflammation, from the nature of the direct action of the medicine, it would, of course, be interdicted; and in such a case, it seems to me, it would be proper to administer it per enema, as suggested by Professor Elliotson.

After having exhibited the quinia in sufficient quantities to arrest the recurrence of the paroxysms, if it be omitted, or continued in inefficient doses, the patient will be found frequently to remain in a state somewhat intermediate between health and illness for an indefinite period. The appetite does not return; muscular strength, and bodily and mental activity are not regained; and, in short, he is not well, without being able to indicate any particular source of suffering or trouble. I have known such a state continue for ten weeks, and vanish in a few days after the proper indications were acted upon. This condition calls for the continuance of the specific, or its exhibition in larger doses. Under such circumstances, a large dose of quinia will oftentimes produce an immediate internal change of sensation, which the patient cannot well describe, but which is not less striking to himself than to the physician—and from this time, the appetite and strength rapidly return.

I have also observed several instances, in which the disease may be said to be present in a latent form. I mean by this a condition which sometimes occurs after exposure in a malarious region, where it is presumed that the amount of morbific influence imbibed by the system, is not sufficient of itself, or by means of collateral influences, to develop either intermittent or remittent fever. This condition is characterized by anorexia, and in general that combination of symptoms which is commonly expressed by that very convenient term "*bilious,*" without any particular evidences of the liver being the organ chiefly affected. Connected with these symptoms, I have remarked a symptom which, when present, appears to me almost pathognomonic, viz:—a thin, moist coat, equally diffused over the tongue, of a remarkable whiteness. I have characterized it, in my case-book, frequently by the term *chalky*. It is not easy to describe the peculiarity, but when I have observed it, without inquiring the history of the patient, I have often, at once pronounced the character of the difficulty.

If the symptoms of these patients be closely inquired into, there will be found irregular variations of sensation as it regards temperature, and also of the circulatory system, which will render the diagnosis more positive; and the immediate and striking benefit derived from the quinia, verifies its correctness.

I will remark in this connection, also, that patients, after having experienced intermittent fever, will sometimes complain of troublesome night perspirations, without any other unpleasant symptoms. This seems to be a sequela of the disease, and is readily relieved by the quinia in doses of five grains at night.

It is of consequence (to what extent I am unable to judge) to connect the views which have been presented relative to the disease under consideration, with the geographical position of the place at which they are written. As the general characters of the climate of this place are probably sufficiently known, I will not lengthen this article by their description. It is highly probable, that from this source are derived influences upon the character of the disease, and the method of treatment, which are of great importance, and differ widely in different localities. This is a subject deserving attentive consideration; and it is much to be desired, that with reference to this point, physicians located in different sections of our country where the disease prevails, would present the results of their experience, so that a comparison might be instituted. Upon this subject, the following remarks of Broussais are valuable. "In the *damp* and *cold* climate of Belgium and Holland, advantageous results were obtained from the immediate administration of bark. At Udine, situated in a *dry*, *hot* country, where I have since practised, I never succeeded in cutting short a single case of fever, from the month of August to the autumn, without observing a succession of those symptoms of inflammatory sensibility of the alimentary canal, of which I have spoken. The climate of Belgium destroys the irritability of the gastric mucous membrane, and enables it to support much stimulation. The atmosphere of Italy being more charged with caloric and electricity, communicates a sensibility to the organs which does not permit the use of irritants."—*Chronic Phlegmasiæ*, vol. i. Translation by Drs. Hays and Griffith.

The tendency to relapse in intermittent fever, after a greater or less period, is universal. Almost invariably, patients who once experience the disease, sooner or later have recurrence of the paroxysms. They are apt, in consequence, to express impatience, and to utter complaints against the remedy which relieves only for a season. This is, to say the least, in bad taste. There ought, on the other hand, to be felt the highest gratitude, that for a disease prevailing so generally over large portions of our country, and which, if not susceptible of relief, would be an almost insuperable obstacle in the development of the resources of our national domains, we have a specific which, whenever it occurs or recurs, will, almost invariably, produce speedy relief.

In this latter point of view, it becomes a matter of immense consequence that the efficacy of this invaluable remedy should be fully known, and that the unreasonable prejudices which exist in the common mind against its use, should be removed. These considerations chiefly have induced me to give the results of my experience, which, I am well aware, has been limited when compared with that of thousands in different sections of our country; and should the facts and views which I have presented, fortunately induce others to publish the results of longer and more extensive observations of a disease, which, (when we consider its intrinsic importance, in connection with unlimited means for its study,) has occupied by far too small a portion of the medical literature of our country; and the etiology, pathology, history, and therapeutics of which are still sadly deficient in definite, established principles, my object will be attained.

BUFFALO, *May* 19*th*, 1841.

[THE above we have abstracted from an admirable paper of Dr. Flint, first published in the American Journal of Medical Sciences, for October 1841.

We give it as much room as the size of our journal will allow, for we could not perhaps select any matter, so appropriate to the wants of the profession, or the prejudices of the people in our vicinity, as that contained in the above paper. We only regret, that we have been obliged to omit some interesting cases; though we have endeavored to preserve entire the deductions and practical remarks founded thereon.—ED.]

